# A Review of Computer Vision-Based Structural Deformation Monitoring in Field Environments

**DOI:** 10.3390/s22103789

**Published:** 2022-05-16

**Authors:** Yizhou Zhuang, Weimin Chen, Tao Jin, Bin Chen, He Zhang, Wen Zhang

**Affiliations:** 1 College of Civil Engineering, Zhejiang University of Technology, Hangzhou 310014, China; yizhouzhuang@zjut.edu.cn (Y.Z.); weiminchen@zjut.edu.cn (W.C.); wenzhang@zjut.edu.cn (W.Z.); 2 School of Engineering, Zhejiang University City College, Hangzhou 310015, China; chenbin@zucc.edu.cn (B.C.); zjuzhanghe@zju.edu.cn (H.Z.); 3 Department of Civil Engineering, Zhejiang University, Hangzhou 310058, China; 4 Yangtze Delta Institute of Urban Infrastructure, Hangzhou 310005, China

**Keywords:** computer vision, structural deformation monitoring, field environment, environmental impact, target tracking algorithm impact

## Abstract

Computer vision-based structural deformation monitoring techniques were studied in a large number of applications in the field of structural health monitoring (SHM). Numerous laboratory tests and short-term field applications contributed to the formation of the basic framework of computer vision deformation monitoring systems towards developing long-term stable monitoring in field environments. The major contribution of this paper was to analyze the influence mechanism of the measuring accuracy of computer vision deformation monitoring systems from two perspectives, the physical impact, and target tracking algorithm impact, and provide the existing solutions. Physical impact included the hardware impact and the environmental impact, while the target tracking algorithm impact included image preprocessing, measurement efficiency and accuracy. The applicability and limitations of computer vision monitoring algorithms were summarized.

## 1. Introduction

Transportation infrastructure systems such as bridges, tunnels and railroads are important component systems for national social production and national development. With the tremendous development of social productivity, these transportation infrastructures are tested in two major ways. On the one hand, the tonnage and number of existing means of transportation may exceed the design load-carrying capacity; on the other hand, civil engineering structures including bridges, are subjected to various external loads or disasters (such as fire and earthquakes) during their service life, which in turn reduces the service life of the structures. By carrying out inspection, monitoring, evaluation, and maintenance of these structures, we can ensure the long life and safe service of national infrastructure and transportation arteries, which is of great strategic importance to support the sustainable development of the national economy.

In the past two decades, structural health monitoring (SHM) has emerged with the fundamental purpose of collecting the dynamic response of structures using sensors and then reporting the results to evaluate the structures’ performance. Their wide deployment in realistic engineering structures is limited by the requirement of cumbersome and expensive installation and maintenance of sensor networks and data acquisition systems [1,2,3]. At present, the sensors used for SHM are mainly divided into contact type (linear variable differential transformers (LVDT), optical fiber sensors [4,5,6,7,8,9], accelerometers [10,11], strain gauges, etc.) and non-contact types (such as global positioning systems (GPS) [12,13,14], laser bibrometers [15], Total Station [16], interferometric radar systems [17], and level computer vision-based sensors). Amongst the existing non-contact sensors, the GPS sensor is easy to install, but the measurement accuracy is limited, usually between 5 mm and 10 mm, and the sampling frequency is limited (i.e., less than 20 Hz) [18,19,20,21,22]. Xu et al. [23] made a statistical analysis of the data collected using accelerometers and pointed out that the introduction of maximum likelihood estimation in the process of fusion of GPS displacement data and the corresponding acceleration data can improve the accuracy of displacement readings. The accuracy of the laser vibrometer is usually very good, ranging from 0.1 mm to 0.2 mm, but the equipment is expensive and its range is usually less than 30 m [24]. Remote measurements can be performed with better than 0.2 mm accuracy using a total station or level, but the dynamic response of the structure cannot be collected [25,26].

With the development of computer technology, optical sensors and image processing algorithms, computer vision has been gradually applied in various fields of civil engineering. High-performance cameras are used to collect field images, then various algorithms are used to perform image analysis on a computer to obtain information such as strain, displacement, and inclination. After further processing of these data, the dynamic characteristics such as mode shape, frequency, acceleration and damping ratio can be obtained. Some researchers extract the influence line [27,28] and the influence surface [29] of a bridge structure from the spatial and temporal distribution information of vehicle loads, which are used as indicators to evaluate the safety performance of the structure. However, the long-term application of computer vision in the field is limited in many ways; for example, the selection of targets, measurement efficiency and accuracy, environment impact (especially the impact of temperature and illuminate changes).

This paper reviews the computer vision-based studies in field environments, including the system composition, target tracking algorithms, environmental influencing factors and current achievements. It is organized as follows: Section 2 briefly describes the basic hardware composition of the computer vision monitoring system; Section 3 introduces the flow of monitoring, camera calibration methods, feature extraction and different target tracking algorithms. Section 4 reviews the current application scenarios of computer vision in the field of SHM, analyzes the shortcomings in field applications and lists the corresponding solutions, and finally briefly describes some basic requirements of long-term monitoring. Section 5 makes a summary and points out the important problems that need to be further studied in long-term field applications of deformation monitoring systems based on computer vision.

## 2. System Composition

A computer vision-based structural deformation monitoring system includes an image acquisition system and an image processing system. The image acquisition system includes a camera, lens, and target to collect video images, while the image processing system performs camera calibration, feature extraction, target tracking, and deformation calculation, which purpose is to process the acquired image and calculate the structural deformation. This section will briefly introduce the basic components of the image acquisition system in the computer vision monitoring system. The image processing system will be introduced in Section 3.

### 2.1. Camera

The camera is an important part of the image acquisition system, and its most essential function is to transform received light into an electrical signal through a photosensitive chip and transmit it to the computer. Photosensitive chips can be divided into CCD and CMOS according to the different ways of digital signal transmission. The main differences between them are that CCD has advantages over CMOS in imaging quality, but its cost is much higher than that of CCD, so it is suitable for high-quality image acquisition; CMOS is highly integrated and saves electricity compared with CCD, but the interference of light, electricity and magnetism is serious and its anti-noise ability is weak, so it is more suitable for high-frequency vibration acquisition [30,31].

The selection of the camera needs to consider the following points: (1) the appropriate chip type and size is to be selected according to the measurement accuracy and application scenario; (2) because of the limited bandwidth, the frame rate and resolution of the camera are contradictory, so the frame rate and resolution should be balanced in camera selection; (3) industrial cameras appear to be the only option for long-term on-site monitoring.

### 2.2. Lens

The lens plays an important role in a computer vision system, and its function is similar to that of the lens in a human eye. It gathers light and directs it ontp the camera sensor to achieve photoelectric conversion. Lenses are divided into fixed-focus lenses [32,33] and zoom lenses [34]. Fixed-focus lenses are generally used in laboratories, and high-power zoom lens are generally used for long-distance monitoring such as of long-span bridge structures and high-rise buildings. The depth of field is related to the focal length of the lens; the longer the focal length, the shallower the depth of field.

The following points need to be considered in the selection of shots: (1) a low distortion lens can improve the calibration efficiency; (2) an appropriate focal length for the camera sensor size, camera resolution and measuring distance should be selected; (3) a high-power zoom lens is appropriate for medium and long-distance shooting.

### 2.3. Target

The selection of targets directly affects the measurement accuracy, and an appropriate target can be selected according to the required accuracy. There are mainly two kinds of target: artificial targets and natural targets. Ye et al. [35] introduced six types of artificial targets [19,36,37] (flat panels with regular or irregular patterns, artificial light sources, irregular artificial speckles, regular boundaries of artificial speckle bands, and laser spots) and a class of natural targets [38,39]. Artificial targets can provide high accuracy and are robust to changes in the external environment, just as artificial light sources can improve the robustness of targets in light and the possibility of monitoring at night. The disadvantage of artificial target is that they need to be installed manually, which may change the dynamic characteristics of the structure. Natural targets rely on the surface texture or geometric shape of the structure, which is sensitive to changes of the external environment, and their accuracy is not high.

The following points should be noted in the selection of targets: (1) when the target installation conditions permit, priority should be given to selecting artificial targets to obtain stable measurement results; (2) the selection of targets should correspond to the target tracking algorithm in order to achieve better monitoring results.

## 3. Basic Process

**Figure 1:**
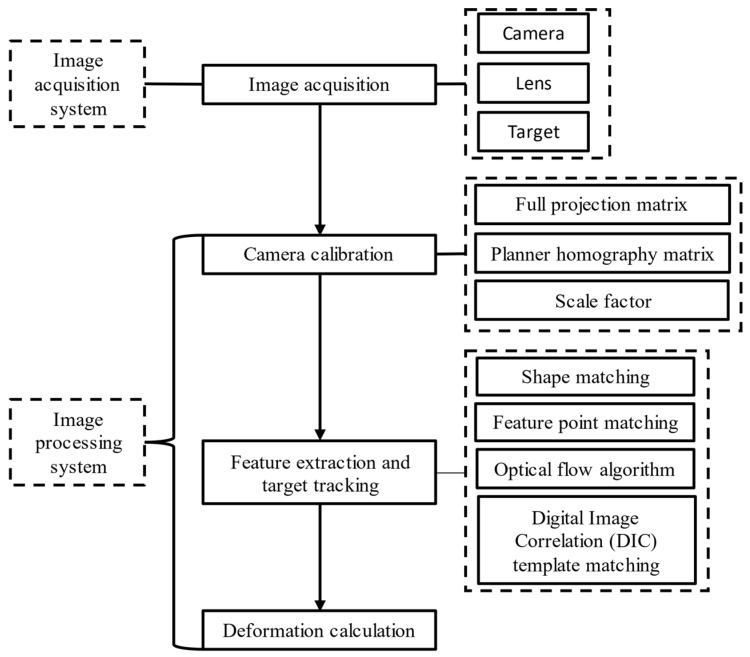
Process of deformation monitoring based on computer vision.

The flowchart of deformation monitoring based on computer vision is shown in Figure 1, and can be summarized as follows: (1) assemble the camera and lens to aim at artificial or natural targets, and then acquire images; (2) calibrate the camera; (3) extract features or templates from the first frame of the image, then track these features again in other image frames; (4) calculate the deformation. The following is a brief description of camera calibration, feature extraction, target tracking and deformation calculation.

### 3.1. Image Acquisition

Image acquisition includes these steps: (1) determine the position to be monitored; (2) arrange artificial targets or use natural targets on the measurement points; (3) select an appropriate camera and lens; (4) assemble the camera lens and set it firmly on a relatively stationary object; (5) aim at the target and acquire images.

### 3.2. Camera Calibration

Camera calibration [40] is the process of determining a set of camera parameters which associate real points with points in the image. Camera parameters can be divided into internal parameters and external parameters: internal parameters define the geometric and optical characteristics of the camera, while external parameters describe the rotation and translation of the image coordinate system relative to a predefined global coordinate system [41]. In order to obtain the structural displacement from the captured video image, it is necessary to establish the transformation relationship from physical coordinates to pixel coordinates. The common coordinate conversion methods are full projection matrix, planar homography matrix, and scale factor.

#### 3.2.1. Full Projection Matrix

The full projection matrix transformation reflects the whole projection transformation process from 3D object to 2D image plane. The camera internal matrix and external matrix can be obtained by observing a calibration board, which can be used to eliminate image distortion and has a high accuracy [42]. Commonly used calibration boards include checkerboard [43] and dot lattice [44].

**Figure 2:**
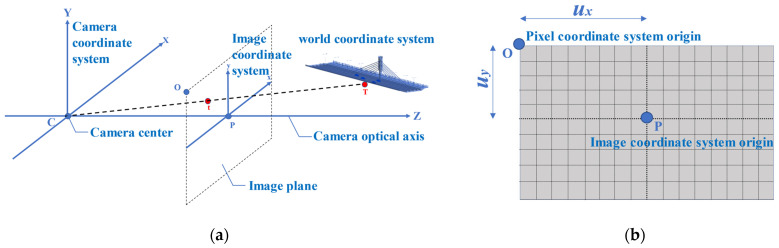
(**a**) Relationship among the camera coordinate system, the image coordinate system, and the world coordinate system; and (**b**) Relationship between the pixel coordinate system and the image coordinate system.

Figure 2a shows the relationship between the camera coordinate system, the image coordinate system and the world coordinate system. A point T (*X*, *Y*, *Z*) in the real 3D world appears at the position t (*x*, *y*) in the image coordinate system after the projection transformation (where the origin of the coordinates is P). The relationship between the pixel coordinate system and the image coordinate system is shown in Figure 2b. Therefore, the equation for converting a point from a coordinate in the 3D world coordinate system to a coordinate in the pixel coordinate system is

(1) $$S\left[\begin{array}{l} x\\ y\\ 1 \end{array}\right]==\left[\begin{array}{cccc} fx & \gamma & u_{x} & 0\\ 0 & fy & u_{y} & 0\\ 0 & 0 & 1 & 0 \end{array}\right]\left[\begin{array}{cc} R & t\\ 0 & 1 \end{array}\right]\left[\begin{array}{c} X\\ Y\\ Z\\ 1 \end{array}\right]=M_{1}M_{2}X$$

where *S* is the scale factor from Equation (3), *f_x_* and f_y_ are the camera lateral axis and vertical axis focal lengths, *γ* is the angle factor of the lens, *u_x_* and *u_y_* are lateral and vertical offsets of the principal axs, respectively, *R* is the rotation matrix of size 3 × 3 and *t* is the translation matrix of size 3 × 1, M_1_ is the camera internal parameter, and M_2_ is the camera external parameter.

Park et al. [45] and Chang et al. [41] calibrated with T-bar and checkerboard respectively to eliminate the measurement error caused by camera distortion and accurately measure the 3D dynamic response of a structure.

#### 3.2.2. Planar Homography Matrix

In practical engineering applications, the above calibration process is relatively complex. To simplify the process, Equation (1) can be expressed

(2) $$S\left[\begin{array}{l} x\\ y\\ 1 \end{array}\right]=\left[\begin{array}{lll} k_{11} & k_{12} & k_{13}\\ k_{21} & k_{22} & k_{23}\\ k_{31} & k_{32} & k_{33} \end{array}\right]\left[\begin{array}{c} X\\ Y\\ 1 \end{array}\right]=K\left[\begin{array}{c} X\\ Y\\ 1 \end{array}\right]$$

where *K* is called planar homography matrix [46], which can reflect the relationship between the corresponding points on two images and is not affected by the angle between the optical axis and the structural plane [43].

The planar homography matrix is suitable for the case where there is an angle between the image plane and the moving plane of the object, and the angle is not easy to measure [42]. The position of at least four known points on the moving plane can be used to solve the planar homography matrix. Khuc et al. [29] and Xu et al. [47,48] both used known structural dimensions to solve for the planar homography matrix, construct the corresponding relationship between image coordinates and 3D world coordinates, and estimate the time history information of lateral and vertical displacement of a bridge.

#### 3.2.3. Scale Factor

**Figure 3:**
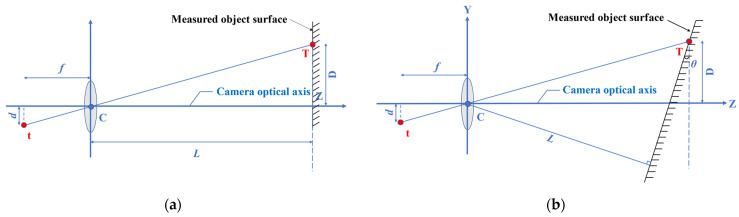
Scaling factor calibration method. (**a**) Camera optical axis orthogonal to measured object surface; (**b**) Camera optical axis intersecting obliquely with measured object surface.

The scale factor (S) provides a simple and practical calibration method. As shown in Figure 3a, when the camera optical axis is perpendicular to the surface of the object, *S* (unit: mm/pixel) can be obtained based on the internal parameters of the camera (focal length, pixel size) and the external parameters of the camera and the surface of the object (measurement distance) in a simplified calculationfrom the simplified formula

(3) $$S=\frac{L}{f}d_{pixel}=\frac{D}{d}d_{pixel}$$

When the optical axis of the camera is not perpendicular to the surface of the measured object (as shown in Figure 3b), the included angle would affect the measurement accuracy [49]. Feng et al. [1] studied the influence of different angles between the optical axis and the surface of the measured object on the accuracy, and found that *S* can be determined by:

(4) $$S=\frac{L_{}}{f\;\cos^{2}\theta }d_{pixel}=\frac{D}{d\;\cos^{2}\theta }d_{pixel}$$

where *f* represents the focal length; *L* represents the distance from the camera to the measured object surface along the optical axis, also known as object distance; *D* represents the distance from the measuring point to the optical axis; and *d* represents the distance from the measuring point on the image to the origin.

References [50,51,52,53,54,55] build *S* according to known physical dimensions on the surface of the object (such as the dimension of an artificial object or the dimension of the structural member obtained from the design drawing) and the corresponding image dimensions to measure the displacement of the structure.

Among these camera calibration algorithms, the appropriate coordinate conversion method needs to be selected according to the field environment and measurement purpose. The full projection matrix and the planar homography matrix have no restraint on camera position but need a calibration plate. The full projection matrix is suitable for 3D deformation monitoring, and the planar single response matrix and scale factor are suitable for 2D deformation monitoring.

### 3.3. Feature Extraction and Target Tracking

Feature extraction is used to obtain the unique information in the image (such as shape features, feature points, grayscale features, and particle features). The purpose of target tracking algorithms is to find these features again in other image frames. Common target tracking algorithms in civil engineering structural deformation monitoring include shape matching, feature point matching, optical flow estimation and digital image correlation (DIC) template matching.

#### 3.3.1. Shape Matching

In an image, shape is a description of an edge or region, and shape matching is an image matching algorithm to identify and locate measured objects through image edge features. There are many algorithms for edge detection, such as Zernike operator [56], Roberts operator, Sobel operator [57], Log operator [58], Canny operator [59] and generalized Hough algorithm [60]. Among them, the Canny operator is widely used because of its high performance [61,62].

The principle of shape matching is relatively simple and can be used for displacement monitoring of structures with obvious shapes. The advantages are: (1) the calculation is relatively simple and the matching speed is fast; (2) it is robust to change of illumination because it tracks the geometric boundary of the object; (3) this measurement has an advantage for linear structures such as slings.

#### 3.3.2. Feature Point Matching

Feature point matching is a target tracking method based on feature extraction and matching. The key points in computer vision are those which are stable, unique and invariant to image transformation, such as building corners, connection bolts, or other shaped targets [63,64]. The common methods of feature point detection include Harris Corner [65], Shi–Tomasi Corner [66], scale invariant feature transform (SIFT) [32,67], speed-up robust feature (SURF) [68], binary robust independent elementary features (BRIEF) [69], binary robust invariant scalable keypoint (BRISK) [70], and fast retina keypoint (FREAK) [71]. 

A feature point matching algorithm needs to select appropriate feature descriptors according to the measurement object to describe feature points mathematically and carry out image registration. It is usually suitable for structures with rich textures or certain shapes (Such as circle, hexagon or rectangle). Feature point matching has the following characteristics: (1) it deals with the whole image area and has accurate matching performance [72]; (2) it extracts texture features of the structure and is not sensitive to illumination and shape transformation; (3) the greater the number of feature points used, the higher is the precision (however, this increases the calculation time).

#### 3.3.3. Optical Flow Algorithm

Optical flow algorithm is an image registration technique in which the surface motion in a three-dimensional environment is approximated as a two-dimensional field by using the spatio-temporal pattern of image intensity [73]. The optical flow algorithm can accurately provide the velocity and displacement of the object by tracking the trajectories of pixels, but it has great limitations and makes the following assumptions [74]: (1) the brightness of objects in adjacent frames remains unchanged; (2) the motion of objects in adjacent frames is small enough; (3) the motion between adjacent pixels is consistent [75]. Common optical flow algorithms include Lucas–Kanade [76,77], Horn–Schunck method [78], Farneback method [79], block match method [80], and phase-based optical flow [45,81,82]. Among those, Lucas–Kanade is fast and easy to implement, and it can perform motion tracking in the selected measurement area, especially of robust feature points, while other algorithms need to calculate every pixel in the image, which is slow.

The optical flow algorithm is similar to the feature point matching algorithm in that it tracks feature points on the image and prefers target patterns with distinct and robust features over the whole test period. The optical flow algorithm has the following characteristics: (1) target features need to be clear; (2) sensitivity to illumination changes; (3) only motion components perpendicular to local edge direction can be detected, such as bridge cable vibration; (4) optical flow describes the motion information of the image brightness and is more suitable for measuring dynamic displacement.

#### 3.3.4. DIC Template Matching

The basic principle of DIC is to compare the same points (or pixels) recorded between two images before and after deformation, and to calculate the motion of each point [83]. As a representative non-interference optical technique, DIC has the advantage of continuous measurement of the whole displacement field and strain field. It is a powerful and flexible surface deformation measurement tool in experiments on solids, and it has been widely accepted and used [84,85,86,87]. If we track only a small pixel area, we can track and monitor the displacement of the measuring points of the structure [88,89], which is called template matching. The basic process of monitoring displacement by template matching is as follows [90,91,92]: (1) select some areas of the first frame image as templates; (2) use these templates to scan line by line in a new image frame; (3) then use the relevant criteria to match the degree of similarity and determine the pixel coordinates of the matched template; (4) calculate the pixel displacement and convert it to the actual displacement. 

The relevant criteria include the following six mathematical algorithms: (1) cross-correlation (CC); (2) normalized cross-correlation (NCC); (3) zero-normalized cross-correlation (ZNCC); (4) sum of squared differences (SSD); (5) normalized sum of squared differences (NSSD); and (6) zero-normalized sum of squared differences (ZNSSD) [93].

In computer vision-based displacement measurement, the NCC matching method is the most popular, and there are numerous applications of the method. Template matching based on DIC has the following characteristics: (1) it is not very robust to light changes, slight occlusions, and scale changes; (2) an artificial target is beneficial to improve the success rate of matching; (3) huge computational expense during the template matching; calculation in the frequency domain can save computation time.

### 3.4. Deformation Calculation

Deformation computation is the process of transforming pixel displacement into actual displacement. First, high quality images are collected; then, 3D motion in the real world is decomposed into planar motion by camera calibration; later, the matching algorithm is used to track and calculate the pixel distance of the target moving in the image plane. Finally, the pixel distance is converted into proportional actual distance.

The accuracy of displacement depends not only on the camera calibration method and target tracking algorithm, but also on the environment, so the influence of environment on the accuracy of displacement calculation needs to be understood. This is the problem that needs to be solved in current field applications. The most important thing is to improve the algorithm so that it can adapt to the changing environment.

## 4. Computer Vision-Based Deformation Monitoring in Field Environment

Computer vision-based sensors have made great strides in the lab, and computer vision-based monitoring systems have the following advantages over conventional attached sensors and other non-contact optical sensors: (1) providing displacement measurements in both time and frequency domains [94]; (2) measuring multiple targets simultaneously [95]; (3) non-contact long-distance high-precision measurement; (4) simple setup and lower labor intensity [96].

Although computer vision-based structural deformation monitoring has broad prospects, there are still some challenges and problems to be studied. Up to now, there have been few cases in which the structural deformation monitoring system based on computer vision could be used stably in structural health monitoring for a long time. In the process of indoor experimental application research, a controllable experimental environment enables the image acquisition system to stably collect high-quality images or video files and to get better results by post-processing. However, in on-site long-term monitoring, the erection conditions of targets and cameras are limited by the on-site environment through factors such as target installation difficulty, environmental vibration, measuring distance, and image acquisition and transmission rate. Image processing also needs to meet the requirements of long-term stable real-time monitoring under uneven changes of temperature and light, occlusion, and real-time processing of image data, and to output reliable displacements. These challenges and problems will be important parts to be considered in future research and engineering practice.

### 4.1. Application Scenario of Computer Vision Monitoring System

Researchers began to use video analysis in civil engineering structure monitoring and achieved results. At present, computer vision-based monitoring technology has been applied to many fields of SHM, including bolt looseness detection [97,98,99]; quantifying disaster impact [100,101,102,103,104]; cable force monitoring [55,105,106,107,108]; modal frequency monitoring [2,19,52,63,74,95]; structural deformation measurement in 2D [34,109,110,111,112] and 3D [50,113]; bridge structural influence line [27,28] and influence surface measurement [29]; structural damage detection and localization [51,52]; updating finite element models of structures [114,115]; static and dynamic rotational angle measurement of large civil structures with high resolution [116], and spatio-temporal distribution of traffic loads [117].

In practice, researchers have found the influence of computer vision-based monitoring system on practical applications. According to the system composition, these influencing factors are divided into two aspects: physical influence research and target tracking algorithm influence research. Physical influence corresponds to the image acquisition system, and target tracking algorithm corresponds to the image processing system. Section 4.2 and Section 4.3 will focus on the impacts of these two aspects, analyze their influence mechanisms, and present some solutions.

### 4.2. Hardware Impact and Environmental Impact

#### 4.2.1. Hardware Impact

The camera and targets are the main parts of the image acquisition system. In field applications, we need to consider the stability of the long-term use of the camera and solve the problem that target cannot be installed on some structures.

- Camera

Shutter mode and photosensitive chip size can lead to a difference in imaging. The rolling shutter method may cause image distortion when recording fast-moving objects. When using this type of camera, the effect of this distortion on the measurement results should be corrected, and a global shutter can solve this problem [110,118]. The bigger the photosensitive chip, and the higher the picture pixel density, the higher the theoretical measurement accuracy, but the higher the economic cost. In addition, camera heating will cause chip heating, resulting in errors. Ma et al. [119] conducted an in-depth study on the strain measurement errors caused by self-heating of CCD and CMOS cameras. When the temperature increases, the virtual image expansion will cause a 70–230 *με* strain error in the DIC measurement, which is large enough to be noticed in most DIC experiments and hence should be eliminated.

The inherent frequency of a structure determines the sampling frequency of the camera. According to Nyquist’s Theorem, when the sampling frequency is less than twice that of the measured signal, aliasing (i.e., false low-frequency components in the sampled data) may occur. Different sampling frequencies should be adopted for different structures: a rigid structure needs high sampling frequency, while a flexible structure can permit a lower sampling frequency. This not only can save energy, but also can reduce calculation and allow real-time monitoring. For example, if the highest frequency at which significant (visibly detectable) motion of the bridge structure occurs is below 10 Hz, then a sampling rate of 20 Hz should be sufficient to avoid aliasing.

- Target

The target is key to the accuracy of computer vision measurement. There are two types of targets: artificial targets and natural targets. The artificial target is an obstacle in the field application of current computer vision-based measurement methods. It must be attached to the surface of the measured object. The installation of artificial targets may require equipment such as a bridge inspection vehicle, which is not only time-consuming, but also unsafe. In addition, the installation of artificial targets may change the dynamic characteristics of the structure [74]. Brownjohn et al. [112] studied the effect of the properties of targets (including both artificial targets and edge features of the structure). The results show that the noise of the vision sensor is inversely proportional to the size of the target. 

Ehrhart et al. [120] attached a circular target to a pedestrian bridge to measure the bridge vibration, and proved that, for a single frame structure and an observation distance within 30 m, a motion larger than 0.2 mm can be detected. Khuc et al. [32,64] proposed a new vision-based displacement measurement method that did not require installation of manual markers and instead used robust features extracted from the image as virtual targets. Fukuda et al. [2] and Ye et al. [121] used feature matching between continuous images to realize displacement measurement. Yoon et al. [38] introduced a target-free approach for vision-based structural system identification using the Kanade–Lucas–Tomasi (KLT) tracking algorithm and Shi–Tomasi corners. This work could accommodate multi-point displacement measurement of a six-story building model in the laboratory; however, it did not provide verification with conventional displacement sensors. Dong et al. [74] extracted virtual markers from images using robust feature detection algorithms that represent texture or other unique surface features of the structure, and can select the best markers according to different scenarios. They thus made the matching algorithm more adaptive, and verified the effectiveness of the algorithm by measuring structural vibrations of soccer stadium bleachers. Kim et al. [105] carried out environmental vibration tests on the Gwangan Bridge in South Korea to measure the sling structure motion without any target to verify the effectiveness of the non-target strategy in the measurement of the dynamic characteristics of bridge hanger cables.

At present, feature point matching and shape matching are mainly used in target-free strategies, because these two methods can effectively utilize original features of the structure and have robustness to illumination variation.

#### 4.2.2. Environmental Impact

When it comes to field monitoring applications, a variety of external environmental factors that are rare in the laboratory, such as temperature change, camera movement caused by environmental vibration, illumination change, and illumination mutation caused by shielding, will lead to the increase of image noise and the decrease of matching accuracy. In order to solve these problems, researchers have made efforts to reduce the systematic errors caused by these factors. This section first classifies these environmental influences according to their error mechanisms and then summarizes the current solutions.

- Optical refraction

**Figure 4:**
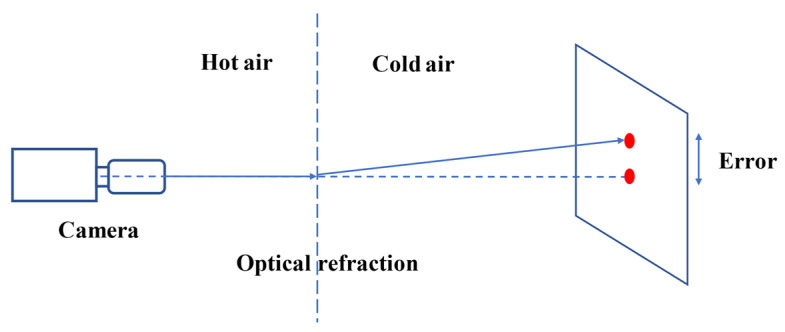
Principle of optical refraction error.

Refraction is a complex optical phenomenon occurring naturally, and vision sensors are easily affected by optical refraction at high temperature, so it is still a challenge to monitor a structure remotely using vision sensors. It can be observed that the change of air density caused by heating causes a change of optical refractive index, which leads to distortion of video images and thus error in displacement measurement. Optical refraction error caused by uneven heating of the air is shown in Figure 4. During a field test, when the air between the camera and the target structure was heated unevenly, measurement error increased as the measurement distance increased [1].

At present, the research on optical refraction mainly focuses on techniques related to static image restoration (that is, processing image distortion). There are many ways to reduce distortion in a single image, such as lucky image, using region-level fusion based on the dual tree complex wavelet transform [122], multi-frame super-resolution reconstruction [123], B-spline-based nonrigid registration [124], and derivative compressed sensing. However, these techniques are custom-made for static images and therefore do not apply to distinguishing structural motion. Luo et al. [125] used a normal random distribution to fit the error caused by optical refraction, which reduces the displacement measurement error caused by optical refraction by about 67.5%. Luo et al. [126] comprehensively studied the characteristics of distortion and displacement error caused by hot air, established a hot air error model, and quantified the measurement error caused by hot air through bridge displacement measurement experiments carried out in high-temperature weather.

Up to now, the influence of optical refraction on vision-based measurement has rarely been mentioned. However, the error caused by optical refraction can reach 50 mm, which shows the importance and necessity of this research. According to Owens’s [127] research, the refractive index of air varies with air pressure, air temperature and air composition. Therefore, when considering the influence of thermal haze, the effects of humidity and air pressure should also be considered.

- Camera motion

**Figure 5:**
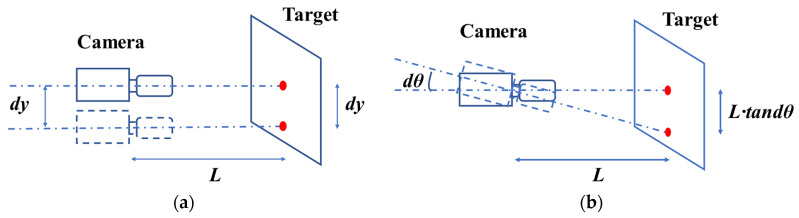
Analysis of error due to camera motion: (**a**) camera translation; and (**b**) camera rotation.

In addition to temperature change, the position of the camera will change due to various influencing factors (e.g., traffic load, thermal expansion and cold shrinkage of brackets, or loose structures). When measuring a real structure outdoors, the position and direction of the camera often change slightly due to wind, vibration and ground instability [128]. Ye et al. [50] believed that the thermal expansion and cold contraction of the mounting bracket would also cause a small change in the position of the camera, and this small motion of the camera, even a very small rotation, would lead to a very large error with increasing range. Figure 5 is a vision-based monitoring system in which the target is fixed, and Figure 5a,b show the errors caused by camera translation and rotation, respectively. It can be seen that, when the camera translates a distance *d_y_* along the vertical optical axis, the error is *d_y_*; However, when the camera rotates slightly through an angle *d_θ_*, the error caused is *L*·tan*d_θ_*, which is unacceptable. Therefore, in order to improve the accuracy of absolute displacement estimation, several camera motion subtraction techniques have been developed: (1) digital high-pass filtering (DHF) [15,129]; (2) background modification (BM) [51,106,130]; (3) inertial measuring unit (IMU) [131]; and (4) ego-motion compensation (EC) [132].

At present, DHF and IMU are mainly used in the field of unmanned aerial vehicle (UAV) displacement measurement, in which DHF can eliminate UAV flight frequencies the by digital high-pass filtering. Garg et al. [15] removed the low-frequency component of the UAS using a high-pass Butterworth filter, and measured the displacement response of a railway bridge under train load; the estimated peak and RMS errors were under 5% and 10%, respectively. An IMU consists of a DC gyroscope and accelerometers that can respond to very low frequency (almost 0 Hz) vibrations. Ribeiro et al. [131] estimated the displacement and rotation of a UAV by numerical integration, and measured a static concrete structure with a peak value error of 1.47 mm (15.5% relative error) and an RMS error of 9.3%.

BM is a simple and convenient method which uses fixed objects such as buildings and mountains as reference points in the background to calculate the relative displacement of the target [51,106,130]. This method is effective to measure the absolute displacement of a structure during the flight of a UAV. Yoon et al. [118] reproduced the vertical dynamic displacement of a pin-connected steel truss bridge undergoing revenue-service train traffic for 250 s in the laboratory using a servo-hydraulic motion simulator. By tracking background characteristics, the motion of the UAV was modified, and the root mean square error of the corrected displacement was reduced from 116 mm to 2.14 mm. Yoneyama et al. [128] measured the plane rotation and translation of a rigid body and changed the camera position and angle. Even if the camera rotation angle is more than 30°, the error is less than 0.1 mm, which verified the feasibility of deducing multi-degree-of-freedom motion of the camera through background correction. Chen et al. [133] measured the antenna at the top of a building with the building itself as a reference point, and measured the relative motion of the antenna. Compared with the frequency measured by a laser vibrometer at close range, the error was less than 1.7%. Khaloo et al. [134] used a distance-based outlier detection method based on Chebyshev’s theorem [135] to accurately estimate the flow vector of pixels in the static background region and subtracted this from the structural pixel flow vector to correct the undesired camera motion.

When the background conditions cannot be satisfied, Lee et al. [132] proposed a long-term displacement measurement strategy that uses a sub-camera to aim at a fixed target near the installation position and calculates the relative motion of the dual-camera system to correct the displacement measured by the main camera. This technical reduced the motion error from 44.1 mm to 1.1 mm.

DHF and IMU may be more suitable for the displacement measurement using drones, because drones always fly with a low-frequency vibration. For long-term monitoring in a relatively stable environment, where there will be no low-frequency vibration, these two techniques have high computational complexity and high cost. Therefore, they are not suitable for long-term monitoring. BM and EC are relatively simple to calculate and have good adaptability to different scenarios, and researchers have carried out field experiments for up to four months to verify their effectiveness [50,132]. These are solutions that can be considered for widespread use.

- Illumination changes and partial occlusion

Optical refraction, camera motion, illumination change and partial occlusion all lead to measurement errors, but the mechanisms are different. Optical refraction and camera motion cause measurement errors, but do not cause changes in image quality, while illumination change and partial occlusion will lead to image quality degradation or even failure to produce a usable image, resulting in the inability to find the target correctly. Deformation monitoring methods based on computer vision are easily affected by environmental conditions when they are applied on a project site [136].

Shi et al. [66] proposed a feature selection criterion based on how the tracker works, which is an optimality criterion based on construction, through which occlusion and point mismatched features can be detected. Yuan et al. [137] proposed a new interpretation standard, which achieves an accuracy of 0.01~0.02 mm under different light intensity conditions and is more accurate than the NCC method. Ullah et al. [138] proposed orientation code matching (OCM), which is based on matching the gradient information around each pixel in the form of an orientation code and is robust to background changes and to illumination fluctuations caused by shadows or highlights. Feng et al. [51,111,114] developed the OCM algorithm into an application program for civil engineering structures. Through indoor shaking table tests, the frequency of a frame structure and the displacement time history of a railway bridge under train load were extracted, and the robustness of the proposed vision sensor to adverse environmental conditions such as low light, background image interference and partial template occlusion was verified. This is of great significance to the development of computer vision in civil engineering displacement monitoring. Luo et al. [139,140] proposed a new edge enhancement matching technique, which can extract both gradient magnitude and gradient direction at the same time, and can track low-contrast features robustly. This technique was verified on a 16.9 m span steel girder railway bridge and a 448 m span steel suspension bridge. Lee et al. [33] firstly used adaptive region of interest (ROI) cropping to narrow the search range, obtained the marker boundary through an edge detection filter, and verified the robustness of the algorithm to illumination by field experiments. Khuc et al. [64] used a geometric transformation method to discard the outliers in the matching pool, so as to reduce the problem of incorrect matching due to frequent illumination changes and monitor the vibration of a stadium stand structure.

In order to overcome the influence of illumination change and partial occlusion, deep learning methods were introduced. Lichao et al. [141] proposed a convolutional neural network structure to learn the adaptive target template update strategy for a given initial template, cumulative template and current frame template. Xu et al. [47] proposed a new algorithm that integrates depth learning, a convolutional neural network and correlation-based template matching. This algorithm covers adjacent regions by changing the size and local movement of the template region and can adapt to drastic changes of the target pattern. It was verified in short-range and long-range monitoring activities, considering background change, illumination change and shadow effects. Dong et al. [142] used spatio-temporal context learning and Taylor approximation to track the target, and verified the robustness under illumination change and fog interference.

Summarizing the above-mentioned methods, there are currently two main types of methods to solve the light transformation and partial occlusion problems: (1) using image gradient magnitude and direction to extract image edge information for target tracking, which is robust to illumination changes and partial occlusion; and (2) using deep learning methods to train the template and background, which can adapt to constant changes in illumination and background.

### 4.3. Impact of Target Tracking Algorithm

Target tracking algorithms directly determine the speed and accuracy of displacement calculation, and depend on the acquisition of high-quality images. The images collected in the field are often accompanied by significant noise (such as Gaussian noise, exponential distribution noise, Rayleigh noise, uniform distribution noise, or salt and pepper noise), and this noise will lead to reduced target tracking accuracy. This section introduces (1) field image preprocessing; (2) real-time performance of the algorithm; (3) accuracy of the algorithm; and (4) the choice of algorithm and how to balance accuracy and efficiency.

#### 4.3.1. Image Preprocessing

The quality of the image directly affects the accuracy of the recognition algorithm, so preprocessing is needed before image analysis. The main purpose of image preprocessing is to simplify the data to the maximum extent so as to improve the reliability of feature extraction, image segmentation, matching and recognition. In field applications, external factors such as sudden changes of illumination, rain, and environmental vibration cannot be controlled. In order to estimate the dynamic characteristics of the structure in this case, image processing technology is especially important [105]. The common digital image processing technology in computer vision includes image transformation, image coding compression, image enhancement and restoration, image segmentation and image description.

In order to correct the geometric distortion between a deformed image and an undeformed image, Kim et al. [14] developed an image processing algorithm which reduces the noise in the frequency domain by natural frequency analysis and accurately measures the dynamic response of a sling. In order to obtain concrete information on steel cantilever beam damage, Song et al. [52] first performed noise filtering by using a discrete wavelet transform, and then provided precise damage localization by using a continuous wavelet transform. Javh et al. [143] combined accelerometer and camera data and used the complex frequency domain least square method to avoid the burden of noise data from the high-speed camera and to measure the modal frequency of a scaled cantilever model. Kim et al. [106] applied spatial image enhancement technology, a smoothing filter and a sharpening filter to reduce image noise and also improve the fuzzy part, which improved the recognition rate of a sling, resulting in a measured cable force error less than 1.1%.

At present, image processing technology has been widely used in the field of vision-based monitoring. However, for different application scenarios, it is an unsolved problem to determine the settings of many parameters (filter, binarization, image pyramid). In addition, excessive image processing requires lengthy calculations, which makes application of image preprocessing techniques to vision-based measurement a challenge.

#### 4.3.2. Measurement Efficiency

Most of the short-term laboratory and field applications are post-processing of recorded images or video files to obtain structural deformation information. On the one hand, the algorithm cannot process the collected image files in real time; on the other hand, it can analyze the saved video files many times to get satisfactory results. While a long-term monitoring system requires real-time and stable output of structural deformation information, the computational efficiency of algorithms can limit the application of vision sensors when high-frequency measurements and simultaneous measurements at multiple points need to be obtained.

Lecompte et al. [144] investigated the effect of the size of a subset of the scatter pattern on the measured in-plane displacement efficiency, and showed that the larger the subset, the higher the efficiency. Guizar-sicairos et al. [87] proposed the upsampled cross correlation (UCC), whose registration accuracy is very accurate for nonlinear optimization algorithms, but greatly reduces the calculation time and memory requirements. Zhang et al. [145] proposed an improved Taylor approximation refinement algorithm and a subpixel localization algorithm, both of which are at least five times faster than UCC. Feng et al. [1,114] limited the search area to the predefined ROI near the template position in the previous image, and then processed the ROI region frame by frame using UCC and OCM algorithms. Through shaking table tests of a frame structure, it was verified that a vision sensor could quickly track the multi-point displacement time history of artificial targets or targets on the structure with a maximum RMS error of 0.72%. Dong et al. [74] used a sparse optical flow calculation method (Lucas–Kanade method), which greatly reduces the number of calculated pixels compared with the global optical flow, and accurately measures the dynamic response of the grandstand structure of a football stadium under crowd load. Guo et al. [146] proposed an improved inverse synthesis algorithm based on Lucas–Kanade, which can complete a displacement extraction in 1 millisecond without the need to install any pre-designed targets on the structure.

In addition to increasing the computational efficiency of the algorithm, improving the hardware configuration can also increase the measurement efficiency, but raises the cost.

#### 4.3.3. Measurement Accuracy

Measurement accuracy is an important indicator of deformation monitoring performance. Unlike indoor measurement, on-site measurement often requires long-distance measurement, and the measurement accuracy decreases with increase of the measurement distance. Under certain hardware conditions, the sub-pixel estimation method can solve the measurement accuracy problem to some extent. Pan et al. [147] summarized subpixel methods, including the coarse–fine search method, double Fourier transform, genetic algorithms, artificial neural network methods, correlation coefficient curve-fitting or interpolation, Newton–Raphson iteration, and gradient-based methods, and pointed out that the latter three methods are the most commonly used due to their simplicity, effectiveness and accuracy. The subpixel method can solve the problem of detection accuracy, and it is necessary to improve the computational efficiency of subpixel accuracy for practical engineering application.

MacVicar-Whelan et al. [148] and Jensen et al. [149] respectively proposed linear interpolation and nonlinear interpolation to improve image resolution, which improves the measurement accuracy to some extent. Bruck et al. [150] proposed a new digital image processing algorithm (Newton–Raphson iteration algorithm), and their experiments show that the Newton–Raphson iteration algorithm can determine displacement and displacement gradient more accurately than a coarse-fine search method. Pan et al. [151] combined an inverse compositional matching strategy with Gauss–Newton without sacrificing subpixel accuracy, and proposed the inverse-compositional Gauss–Newton (IC–GN) algorithm, which is 3 to 5 times faster than the Newton–Raphson iterative algorithm, with an accuracy of less than 0.0222 pixels in the x and y directions. Tian et al. [93] used an efficient and accurate IC–GN algorithm to track a target point, monitored multi-point displacement on a steel truss highway railway bridge, and achieved an accuracy of 0.57 mm at 288 m. Qu et al. [152] proposed an edge detection method combining a pixel level method (Sobel operator) and a subpixel level method (Zernike operator), which is much faster than the Zernike operator, but the detection accuracy is close to that of the Zernike operator. Zhang et al. [145] integrated improved Taylor approximation refinement and localization refinement into vision-based sensors and measured the vibration of a high-speed railway noise barrier. These two improved algorithms (Taylor approximation refinement: RMS error 0.61%, localization refinement: RMS error 0.73%) are at least 5 times faster than traditional UCC (RMS error 0.75%) when the accuracy is similar. Dong et al. [42] combined SIFT feature point and Visual Geometry Group (VGG) descriptor (SIFT–VGG) algorithms as a strategy for vision-based displacement measurement. This integrated strategy improves the measurement accuracy of the original SIFT method by 24% and greatly improves the accuracy of displacement recognition. Fukuda et al. [19] used the azimuth obtained by bilinear interpolation to achieve sub-pixel resolution when measuring structural vibration (vibration frequency: 0.1 Hz~50 Hz, vibration amplitude: 50 mm), with the standard error reduced from 0.14 mm to 0.043 mm. Feng et al. [1] used different levels of subpixel accuracy and pixel accuracy comparison in the laboratory, proved that there is a linear relationship between subpixel accuracy and subpixel level, and accurately measured structural vibration of less than 1 mm. Mas et al. [153] proved the realistic limit of sub-pixel accuracy through a simple numerical model, and found that the maximum resolution enhancement and dynamic range of the image can be achieved.

In these studies, it is found than subpixel can make the measurement accuracy reach a higher level. Many studies have shown that subpixel accuracy varies on the order of 0.5 to 0.01 pixels [147,153]. In theory, subpixel algorithms can achieve the maximum accuracy of displacement calculation, but in practical applications, where real experimental images may be contaminated by many factors such as environmental vibration, temperature change, camera heating, and illumination change, the accuracy often fails to reach ideal levels. In addition, few reported quantitative works have been performed to systematically evaluate their subpixel registration accuracy and computational efficiency or have attempted to solve an existing discrepancy. It is necessary to understand the limitations and performance of these sub-pixel registration algorithms.

#### 4.3.4. Suggestions for Field Application Algorithms

Reviewing the development of application research and engineering practice of SHM based on computer vision, we find it has made great progress. Until now, computer vision has been continuously developed and applied to various fields of civil engineering monitoring. In the application of structural health monitoring based on computer vision, target tracking is the most important step, which directly determines the efficiency, accuracy and reliability of vision sensors. However, the performance of target tracking algorithms depends on application scenarios, and researchers have found applicability and limitations of different algorithms in engineering practice. Table 1 summarizes the advantages and limitations of these algorithms and lists their application scenarios.

### 4.4. Measurement Results

Table 2 classifies the problems from Section 4.1, Section 4.2 and Section 4.3 and presents the current solutions and the results achieved in practical applications. However, these measurement results were determined in specific environments, and actual measurement results must be determined according to the camera resolution, measurement distance, lighting environment and target contrast used in the field.

### 4.5. Other Impacts

An SHM system requires long-term stability in order to assess the state and performance of a structure. The displacement measurement methods in the literature mainly focus on short-term measurements of up to several hours. For long-term measurements of up to several months or even years, the uncertainty and reliability of the computer vision system still need further research. In addition, data acquisition, data transmission and data processing in computer vision systems are new challenges and require highly specialized personnel during and after equipment installation and maintenance, which will require new resources.

When multiple vision-based displacement measurement subsystems need to work together or to measure the displacement of multiple measurement points at the same time [158], the problem of time synchronization of multiple cameras must be solved. Luo et al. [159] developed a vision-based synchronization system using master/slave systems for wireless data communication in order to simultaneously measure multiple points of the structure. Fukuda et al. [2] developed a time synchronization system that connected multiple displacement measurement subsystems using a local area network, enabling computers to communicate with each other using the TCP/IP protocol. Dong et al. [74] synchronized all cameras and potentiometers using an NI Multifunction I/O Device to ensure that multiple cameras and sensors worked synchronously.

## 5. Conclusions and Prospects

This paper briefly describes the composition of computer vision monitoring systems, introduces the basic monitoring process and methods, and pays special attention to the problems and solutions encountered in the application of computer vision in the field environment. From the examined articles, the following main conclusions can be made:

(1) At present, the main application of computer vision in the field of SHM is still focused on the measurement of displacement time history curves of scale models under static and dynamic loading in controlled conditions and for short terms.
(2) A large number of experimental tests and short-term field tests promote the formation of the basic framework of computer vision deformation monitoring systems, and existing research has focused on improving the applicability and stability of image processing algorithms.
(3) Structural deformation monitoring systems based on computer vision have had some solutions to cope with individual external influences (such as target installation difficulty, illuminate change, camera movement and climate transformation). The accuracy and reliability of computer vision-based structural deformation monitoring has made great progress and is gradually approaching practical long-term monitoring.

It has been more than 30 years since computer vision was first applied to civil engineering structural measurement. Vision based sensors have made great progress and achievements in technology, but they still face some limitations and challenges. In the future, we need to do more in these aspects: (1) devise simpler programs or devices to promote their long-term applications in practical engineering; and (2) uncertainty evaluation of vision sensors in long-term applications.

## Figures and Tables

**Table 1:** Characteristics, limitations and application scenarios of the proposed tracking algorithms.

Algorithms	Characteristics and Restraints	Application Scenarios
Shape matching	High efficiency, real-time monitoring, robust properties to non-uniform illumination and partial edge blur, distinct geometry features; short-distance monitoring	Cable structure, tower, long-span bridge, stadium
Feature point matching	High efficiency, high accuracy, robust to illumination change, distinct geometry features; rich surface texture, uncertain number of feature points, short-distance monitoring	Stadium structure, footbridge, railway bridge, urban bridge
Optical flow algorithm	Full field displacement, natural target, suitable for motion tracking; short-distance monitoring, sensitive to illumination change and partial occlusion	Stadium structure, footbridge, railway bridge, urban bridge
DIC template matching	Long-distance and short-distance monitoring; artificial target, sensitive to illumination change	Cable structure, long-span bridge, stadium structure, footbridge, railway bridge, urban bridge, tower

**Table 2:** Measurement results.

Research Point	Reference	Algorithms	Test Description	Results
Target	Ehrhart et al. [120] (2015)	Shape matching (artificial target)	Shaking table test, least squares fit of ellipse, precision quantitative evaluation of accuracy	At the distance of 6 m and 31 m, the error is less than 0.01 mm and 0.2 mm, respectively
	Tian et al. [93] (2016)	DIC template matching (artificial target)	Field test, DIC technology based on IC-GN, displacement~time history curve	At the distance of 300 m, the average error is 0.5674 mm
	Feng et al. [115] (2015)	Template matching (natural target)	Railway bridge test, modal identification, modify finite element model	At the distance of 9 m, the vision sensor and the accelerometer measure the exact same first-order frequency
	Khuc et al. [64] (2017)	Feature point matching (natural target)	Stadium grandstand structure test, feature extraction with Hessian matrix, dynamic displacement measurement	At the distance of 3 m and 13 m, the error is less than 0.01 mm and 0.04 mm, respectively
	Khuc et al. [154] (2020)	Feature point matching (natural target)	Tower test, Canny edge detection and Hough transform, modal identification	At the distance of 1.84 m, first-order natural frequency error below 2%
Camera motion and optical refraction	Garg et al. [15] (2019)	Digital high-pass filtering	Shaking table test, dynamic displacement measurement	At the distance of 4 m, the maximum error is between 10~15%, and the RMS error is between 2~5%
	Ye et al. [50] (2021)	Background modification	Long-term field monitoring, 3D structural deformation measurement	Eliminate the error caused by thermal expansion and cold contraction of camera bracket
	Lee et al. [132] (2020)	Ego-motion compensation	Long-term field monitoring, displacement measurement	Measurement error is reduced from 44.1 mm to 1.1 mm
	Ribeiro et al. [131] (2021)	Inertial measuring unit	Experiment test, modal identification, dynamic displacement measurement	The maximum error of displacement measurement is 1.47 mm and RMS error is 9.3%
	Luo et al. [125] (2020)	Adaptive optical-turbulence error filter	Field test, displacement~time history curve	Measurement errors are significantly reduced by about 67.5% from 0.0845 to 0.0275 mm
Illumination change and partial occlusion	Ribeiro et al. [21] (2014)	Artificial light source	Field test, using artificial light source, displacement ~ time history curve	At the distance of 15 m and 25 m, the error is less than 0.1 mm and 0.25 mm, respectively
	Feng et al. [111] (2015)	A matching algorithm based on the gradient information	Field test, a matching algorithm based on the gradient information, dynamic response of steel and concrete bridges under train load	At the distance of 30.48 m, the maximum displacement error is 2.83%, and the average error is 1.39%
	Shan et al. [155] (2015)	Shape matching	Cable force measurement, shape matching, cable modal identification	The first three frequencies of free vibration of stayed-cable model are accurately measured
	Dong et al. [142] (2019)	A matching method based on Spatio-Temporal Context Learning	Experiment test, a matching method based on Spatio-Temporal Context Learning, illumination change and fog interference, displacement~time history curve	The proposed subpixel estimation method is faster than UCC by about 50 times
	Xu et al. [47] (2021)	Combined with deep learning	Field test, displacement~time history curve	Centimeter-level accuracy can be achieved at distances of more than 715 m
Image preprocessing	Kim et al. [105] (2013)	Image transform technology	Ambient vibration tests, suspension bridge hanger cables, dynamic response and modal frequencies	The error of measuring sling modal frequency and cable force is within 0.5%
	Kim et al. [106] (2013)	Image enhancement techniques	Ambient vibration tests, smoothing filter and sharpening filter, stay cables, dynamic response	The error of measuring suspension bridge hanger cables natural frequency and cable force is within 2%
	Tian et al. [62] (2019)	Image description and segmentation technology	Impact test, Hough transform based on gradient, modal parameters identification	The recognition rate of vibration mode is more than 84%
Measurement efficiency and accuracy	Qu et al. [152] (2005)	Edge detection method using Sobel-Zernike moments operator	numerical tests	The accuracy reaches 87.75% of the sub-pixel level, and the speed is increased by 5 times
	Pan et al. [156] (2011)	DIC template matching based on Newton-Raphson algorithm	Experimental verification, full filed deformation measurement	Without any loss of measurement accuracy, the calculation speed is increased by 120~200 times
	Pan et al. [151] (2013)	DIC template matching based on IC-GN	Numerical tests and experimental verification	The proposed IC-GN is 3~5 times faster than Newton-Raphson
	Zhang et al. [145] (2016)	Integrate two efficient subpixel level motion extraction algorithms	Experimental verification, Taylor approximation refinement algorithm and the localization refinement algorithm, dynamic vibration analysis	In the case of similar accuracy, it is at least 5 times faster than the traditional UCC method (RMS error 0.75%)
	Xu et al. [157] (2019)	Fuse the vision-based displacement measurement with acceleration data	Field test, short-span railway bridge, displacement ~ time history curve	The RMS of measurement noise at the camera-to-target distance of 6.9 m is less than 0.2 mm

## Data Availability

Data sharing is not applicable.
